# Adjuvant Effect of *Quillaja saponaria* Saponin (QSS) on Protective Efficacy and IgM Generation in Turbot (*Scophthalmus maximus*) upon Immersion Vaccination

**DOI:** 10.3390/ijms17030325

**Published:** 2016-03-02

**Authors:** Yujuan Wang, Xiuhua Wang, Jie Huang, Jun Li

**Affiliations:** 1 Yellow Sea Fisheries Research Institute, Chinese Academy of Fishery Sciences, Qingdao 266071, China; 15865563261@126.com (Y.W.); huangjie@ysfri.ac.cn (J.H.); 2 Laboratory for Marine Fisheries Science and Food Production Processes, Qingdao National Laboratory for Marine Science and Technology, Qingdao 266071, China; 3 Key Laboratory of Experimental Marine Biology, Institute of Oceanology, Chinese Academy of Sciences, Qingdao 266071, China; 4 School of Biological Sciences, Lake Superior State University, Sault Ste. Marie, MI 49783, USA

**Keywords:** *Quillaja saponaria* saponins, adjuvant, vaccination, *Scophthalmus maximus*, relative percent of survival (RPS)

## Abstract

The adjuvant effect of *Quillaja saponaria* saponin (QSS) on protection of turbot fry was investigated with immersion vaccination of formalin-killed *Vibrio anguillarum* O1 and various concentrations of QSS (5, 25, 45 and 65 mg/L). Fish were challenged at days 7, 14 and 28 post-vaccination. Significantly high relative percent of survival (RPS) ((59.1 ± 13.6)%, (81.7 ± 8.2)%, (77.8 ± 9.6)%) were recorded in the fish that received bacterins immersion with QSS at 45 mg/L, which is comparable to the positive control group vaccinated by intraperitoneal injection (IP). Moreover, a remarkably higher serum antibody titer was also demonstrated after 28 days in the vaccinated fish with QSS (45 mg/L) than those vaccinated fish without QSS (*p* < 0.05), but lower than the IP immunized fish (*p* < 0.05). Significant upregulation of IgM gene expression has also been identified in the tissues of skin, gill, spleen and kidney from the immunized fish in comparison to the control fish. Taken together, the present study indicated that QSS was able to dramatically evoke systemic and mucosal immune responses in immunized fish. Therefore, QSS might be a promising adjuvant candidate for fish vaccination via an immersion administering route.

## 1. Introduction

Fish aquaculture is expanding rapidly, and is the fastest growing protein-producing sector in the world. However, massive mortalities caused by various infectious diseases have been the most important barrier to the rapid growth and sustainability of intensive fish aquaculture worldwide [1,2]. Fish vaccination has become an established and cost-effective method of controlling certain infectious diseases in industrial fish aquaculture in recent decades [3,4]. Fish vaccines are environmentally friendly biological products, such that application of fish vaccines has successfully reduced the indiscriminate use of antibiotics or other veterinary drugs. This has led to the decrease of antibiotic resistant strains in the aquatic environment, as well as a reduction in the harmful chemical residues in the seafood products [4]. Vaccines delivered via intraperitoneal injection (IP), often in combination with oil-based adjuvants (water-in-oil emulsions), are the most popular approach for fish vaccination due to their superlative protection in comparison to bath/immersion vaccination [5]. However, the extensive labor cost and side effects to the fish are unacceptable. Alternatively, immersion/bath provides a desirable way for fish vaccination given its convenience, safety and low labor cost. However, lower protection of immunized fish in response to some pathogens especially those antigens with insufficient immunogenicity has limited its broad applications in practice of fish aquaculture [6]. Adjuvants have been found to significantly enhance and improve immune responses by eliciting both the humoral and cellular immunity of immunized animals against those weak immunogenic antigens [7]. Thus far, fewer adjuvants have been available for fish vaccination, especially via the immersion/bath route, so the development of novel and more effective adjuvants for fish vaccine delivered by immersion/bath is a worthy and urgent endeavor.

Saponins are a group of chemically heterogeneous steroid and terpenoid glycosides generated by multifarious wild plants or cultivated plants [8,9,10,11,12], some lower marine animals and bacteria [13,14]. These compounds have biological properties specific to their characteristic molecular structures [9], but their functions might vary with different plants resources where the saponins were extracted [15,16,17,18]. Previous studies have revealed that saponins have the immunostimulating effects in animals, and can enhance macrophage phagocytosis, antibody secretions, and the production of cytotoxic T-lymphocytes (CTLs) against exogenous antigens [18,19,20,21,22]. QSS, a mixture of soluble saponins extracted from the bark of the South American tree *Quillaja saponaria* Molina, has become the most potent of immunological adjuvants and commonly used as feed additives in veterinary vaccines [23,24,25]. In regard to aquatic animals, saponins have also demonstrated their immunostimulatory and immune-modulatory effects on innate immune responses in shrimp and in fish, as well as their effects of promoting fish growth [26,27,28,29]. However, most saponins are unstable in aqueous condition and have a seriously hemolytic toxicity to fish at high concentrations [16,17]. Investigations of QSS as a potential adjuvant for applying fish vaccination to enhance the humoral antibody responses, and their diverse effects on fish physiological and immunological functions, are therefore of considerable interest and relevance. The objectives of this study were: (1) to elucidate the protective efficacy of QSS as adjuvant for applying fish vaccination against *Vibrio anguillarum* under different immunizing strategies; and (2) to evaluate the immunostimulating effect of QSS on humoral IgM responses against inactivated *V. anguillarum* bacterins in the cultured turbot upon immersion vaccination.

## 2. Results

### 2.1. Challenge Experiments

Turbots from various immunized groups and their corresponding controls were challenged against pathogenic *V. anguillarum* at days 7, 14 and 28 post-immunization. Cumulative mortalities are shown in Table 1. The fish in the group IP-V that received IP vaccination showed the lowest overall mortalities among all of the groups, whereas the fish that received pretreatments in different concentrations of QSS solutions (especially QSS45) followed by immersion vaccination with *V. anguillarum* bacterins exhibited significant lower mortalities in comparison to those fish immersed only in related QSS solutions or seawater.

**Figure 1:**
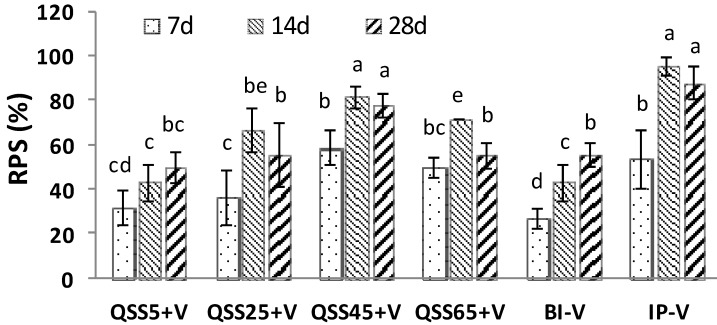
RPS (%) in each group on days 7, 14 and 28 post-vaccination challenged with pathogenic *V. anguillarum.* Significant differences (*p* < 0.05) in RPS among various groups were indicated by different letters. Data represent the mean value ± S.E. of three replicates.

Regarding the protections, at day 7 post-vaccination, the highest RPS value was (59.1 ± 13.6)% in the group of QSS45 + V, and it was even a little bit higher than that of group IP + V (*p* > 0.05) (Figure 1). When the fish were challenged with *V. anguillarum* at the 14th and 28th day post-vaccination, the fish in the group IP + V showed the best protection with the highest RPS values ((95.8 ± 7.2)% and (87.8 ± 13.0)%, respectively) among all immunizing groups. The trial of incorporation of QSS45 pretreatment with bath vaccination (group QSS45 + V) also gained a similar higher protective efficacy ((81.7 ± 8.2)% and (77.8 ± 9.6.0)%, respectively) to IP immunization (group IP + V), which indicated a perfect adjuvant property of QSS in the practice of bath vaccination (Figure 1). However, pretreatment with lower doses of QSS (such as QSS5 + V) could not achieve a satisfactory protection (Figure 1).

### 2.2. Antibody Titers

**Figure 2:**
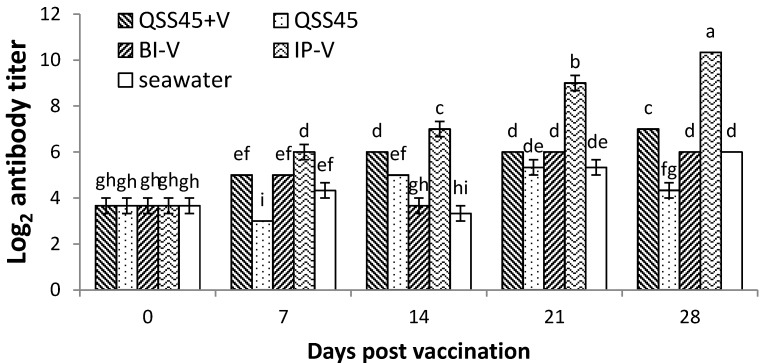
Specific antibody titers against *V. anguillarum* post-immunization in each group. Data represent as the mean value ± S.E. of three replicates. Significant differences (*p* < 0.05) among groups were indicated by different letters.

Serum antibody titers of the fish in group QSS45 + V, as well as the fish in the immunized groups of IP-V, BI-V, and related fish in negative control groups of QSS45 alone and seawater, were analyzed by ELISA at days 0, 7, 14, 21 and 28 post-immunization (Figure 2). The fish vaccinated by IP showed the highest antibody (IgM) titers with an increasing trend following the time post-vaccination, while relatively higher antibody titers were also identified in the fish that received immersion immunization in the presence (QSS45 + V) or absence (BI-V) of QSS pretreatment. No significant difference was found between group QSS45 + V and BI-V, but both were significantly higher in comparison to the fish only immersed in QSS45 or seawater (Figure 2).

### 2.3. Expression of IgM mRNA in Tissues

**Figure 3:**
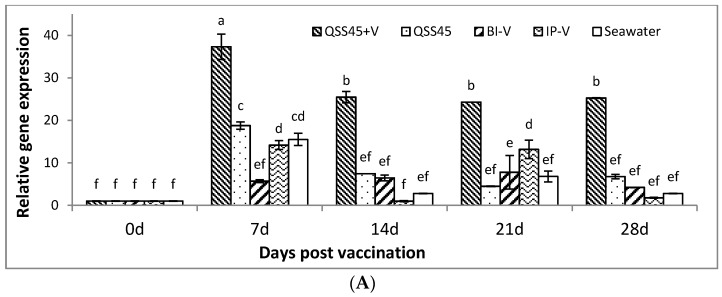

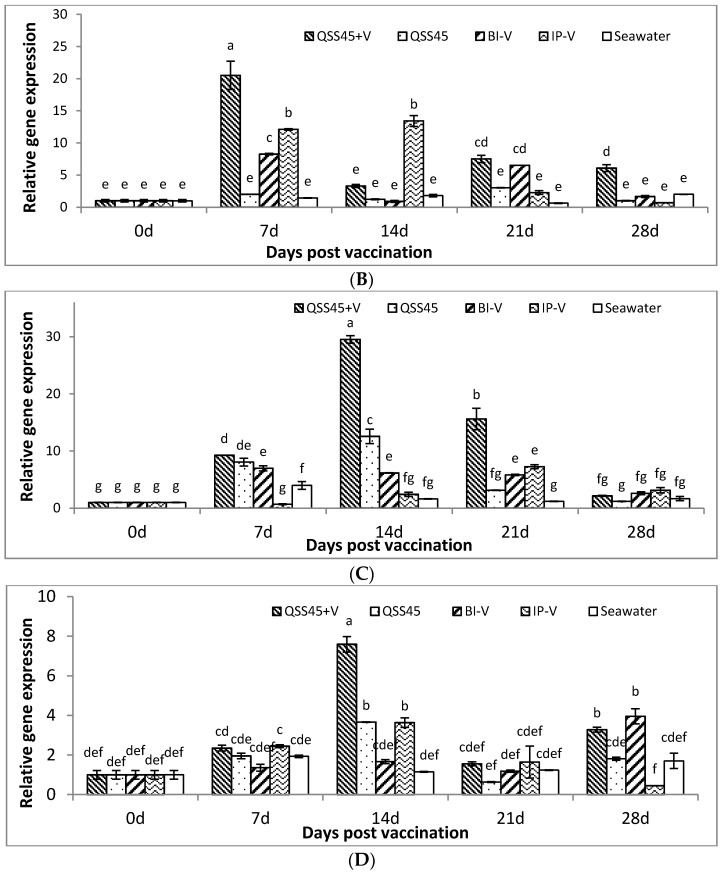
Expression of IgM mRNA in the tissues of skin (**A**); gill (**B**); spleen (**C**) and kidney (**D**) in various treatment groups at days 0, 7, 14, 21 and 28 post-immunization. Different letters indicate significant difference (*p* < 0.05) between groups. Data represent as the mean value ± S.E. of three replicates.

IgM gene expressions in the skin, gill, spleen and kidney of vaccinated and control fish from different groups were evaluated by real-time quantitative PCR. As shown in Figure 3, the highest upregulations of IgM mRNA transcription were apparent in the vaccinated group of QSS45 + V, in particular, 37.3-fold increase in skin and 20.5-fold increase in gill at day 7 post-vaccination, which was significantly higher than those vaccinated groups of BI-V and IP-V, and negative control groups of QSS45 and seawater as well (*p* < 0.05) (Figure 3A,B). Regarding skin, IgM gene expression showed a slight decline after 7 days but still kept a higher level in the group of QSS45 + V at days 14, 21 and 28 (*p* < 0.05). In contrast, no significant elevations of IgM gene expression were observed in the skin samples from other groups including seawater, QSS45, BI-V and IP-V at days 0, 14 and 28 post-vaccination (Figure 3A). Clearly, all vaccinated groups showed significantly higher elevation of IgM gene expression in the gills at day 7 post-vaccination than those negative control groups of QSS45 and seawater (*p* < 0.05). However, the fish that received IP-V showed similar upregulation of IgM gene expressions at days 7 and 14, but remarkably higher than those in group QSS45 + V and the other 3 controls at day 14. Then IgM gene expressions were declined or kept lower level except the fluctuating increase in the groups of QSS45 + V (at days 21 and 28) and BI-V (at day 21) compared to the other control groups (Figure 3B).

In response to the vaccination trials, significant upregulating expressions of IgM gene in spleen were observed at day 7 post-vaccination, in all groups (QSS45 + V (9.26), QSS45 (8.1), BI-V (7.0)) except the group of IP-V, and reached to the highest expression level (29.5-fold increase) in groups of QSS45 + V at day 14 post-vaccination. This was followed by a slight decline at day 21 (15.6-fold increase) and dropping back to the baseline level at day 28. In contrast, group IP-V did not start to increase until day 14 and reached the peak (7.2-fold) at day 21, then decreased to 3.14-fold on day 28 (Figure 3C). Similarly, slight upregulations of IgM gene transcription were detected in kidney at day 7 post-vaccination, and the highest expression appeared in group QSS45 + V at day 14 (Figure 3D). The expressions of IgM gene in group IP-V increased from 1-fold at the beginning to 3.6-fold at day 14, then dropped to 0.45-fold at day 28. In group of BI-V, the expression of IgM gene kept gradually increasing from 1-fold at day 0 to 3.9-fold at day 28. Interestingly, relative lower IgM gene expression levels in the negative control group QSS45 was present in both spleen and kidney during the whole period with the exception of significant elevation at day 14 (Figure 3C,D).

## 3. Discussion

For over 90 years, adjuvants have been used in human vaccines to enhance the immunogenicity of highly purified antigens (such as recombinant proteins/peptides, plasmid DNA, *etc.*) that have insufficient immunostimulatory capacities [7]. Many different adjuvants, including mineral salts (aluminum), water-in-oil emulsions (Freund’s adjuvants), as well as other microorganisms and plants derived components, *etc.*, have been successfully used in human and veterinary animal vaccinations by promoting stronger and more sustainable humoral antibody responses, as well as activating effective cell-mediated immunity [7,30]. Similar effective adjuvants have also been applied in fish vaccination by eliciting faster and stronger protective immune responses against infectious diseases in farmed fish [3,4,31].

Many phytocomponents, such as saponins extracted from various medicinal herbs, were found to enhance mammalian immune system when added to the existing vaccine formulations [9,10,11,32]. QSS was found to be able to block rotavirus infection by inhibiting virus-host attachment through destruction of cellular membrane proteins and/or virus receptors [19]. QS-21, a purified fraction from QSS, was demonstrated a promotion of both systemic and mucosal immune responses against human immunodeficiency virus type 1 [33]. In our previous study, we demonstrated that the innate immune responses of turbot could be significantly enhanced by immersing the fish in subtoxic QSS solutions [28]. The present study clearly shows the significantly higher protections and specific antibody titers in the fish that received a pretreatment in subtoxic QSS solution and was followed by an immersion vaccination in inactivated *V. anguillarum* bacterins solution. These findings suggest that QSS could be used as an adjuvant in fish vaccination by immersion and effectively activate the innate immunity (e.g., complement activation and macrophage phagocytosis) and a consequent antigen presenting activity, thereby initiating the downstream humoral adaptive immune responses of the immunized fish.

Previous studies have revealed that the adjuvant activities of saponins may vary by their different unique molecular structures [9,34]. For example, Sun [35] found that saponins adjuvant activities were affected by the number, length and position of side sugar chains, and the type of glucosyl group of protopanaxatriol-type saponins. Oda [34] also reported that the adjuvant effects of QSS were highly correlated to the high hydrophile-lipophile balance (HLB) value of its structural pen-tacyclictriterpenoids. The normonoterpene moiety of QS-21 was also found to contribute to the induction of cytotoxic effects of CD_8_^+^ lymphocytes [30]. QSS used in the present study is a mixture of soluble triterpene glycosides purified from the bark of *Quillaja saponaria*; therefore, all fractions contained in the commercial saponin product are related to the immunological adjuvant effects of QSS in this study. Individual contributions of each fraction on the immunological responses and related diverse effects in turbot will require further studies.

For fish vaccination, different delivery routes may result in highly variable protective efficacy. IP has been considered to be the best method for immunization because it offers higher protection than other vaccinating strategies such as immersion/bath and oral delivery [36]. In the present study, the fish vaccinated by immersion in the trial of “QSS 45 + V” gained a similar protection to the IP vaccinated fish (*p* > 0.05) against virulent *V. anguillarum* challenge at days 14 and 28 post-vaccination, and even higher protection at day 7 post-vaccination (*p* > 0.05) (Figure 1). A similar earlier antibody response (Figure 2) and higher IgM gene expressions in various tissues were also identified in the same group of fish (Figure 3). All of the findings indicated that QSS at the subtoxic dose of 45 mg/L should be an ideal adjuvant applying in turbot vaccination via immersion by promoting earlier and faster protective immune responses.

Various leukocytes (macrophages, B cells and T cells) exist in fish lymphoid-related tissue such as head kidney, spleen, gill and intestine, and are responsible for acquiring and responding to vaccinating antigens on different delivery routes. Vaccines delivered via direct IP injection will mainly induce the systemic immune responses in spleen and head kidney, while vaccines delivered via immersion/bath or oral routes mainly result in local mucosal immune responses due to the first exposure of antigens at the sites of mucosal tissues such as skin and gill (immersion/bath) or intestine (oral) [3,4]. The stimulation of specific antibody production and upregulation of related immune genes (such as the IgM gene) has been demonstrated in both systemic and mucosal immune responses post-vaccinations [37,38,39,40]. In the skin of teleosts, IgM can be transferred across mucosal epithelia to the outer mucus layer via the polymeric Ig receptor [39]. A previous study indicated that stimulating antibodies appeared faster but in shorter duration in skin mucus than those in serum after immersion/bath vaccination [41]. In our present study, the IgM antibodies were undetectable in the collected skin mucus samples (data not shown). The transcription of skin IgM gene was not significantly induced in the fish that received a single bath immunization (BI-V) of inactivated *V. anguillarum* bacterins; however, remarkably high IgM gene expressions were detected in the skin and gills in the fish that were vaccinated by the strategy of “QSS45 + V.” The mucus IgM in the fish from group “QSS45 + V” was probably digested by hydrolytic enzymes and proteases in the mucus samples that were not completely inhibited.

In this study, remarkable elevations of IgM transcriptions were identified at day 7 in the skin and gill, while the peaks of IgM expression were detected in spleen and head kidney at day 14 post-vaccination. Similar findings have been reported in other fish species, where the highest expressions of IgM was detectable after 1 week in the gills, and after 3 weeks in the spleen and head kidney post-infection or vaccination [41,42]. However, in our present study, the highest IgM gene expression appeared earlier in the skin and gill than those in the spleen and head kidney, which indicates that the antigens were first encountered and processed by leukocytes in the skin and gills, and thereafter antigenic information was transferred to spleen and head kidney post-immersing vaccination. In addition, skin and gills were the major sites of antigen uptake after immersion vaccination, and only small amounts of antigens could be transported to the spleen and the head kidney in fish [6,40,43,44]. Our results showed that pretreatment in subtoxic dose of QSS could improve the antigen absorption by gill and skin, which may subsequently trigger the antigen uptake and presentation by the leukocytes as well as the following adaptive immune responses.

## 4. Materials and Methods

### 4.1. Fish

Disease-free turbots with an average weight of 8.6 ± 1.5 g were purchased from a local fish farm in Yantai, China. The fish were randomly divided into 12 round glass fiber reinforced plastics tanks (1 m^2^ × 1.2 m) with running filtrated seawater (pH 7.8, DO > 6.0 mg·L^−1^, and salinity 28) at 17 °C and fed with commercial pellet food. Fish were acclimatized to laboratory conditions for two weeks prior to experiments.

### 4.2. Saponins

Saponins extracted from the soap bark tree *Quillaja saponaria* were purchased from Alfa Aesar, America (Ward Hill, MA, USA).

### 4.3. Bacterial Strains

A bacterium of *V. anguillarum* MN serotype O1 was isolated from turbot (*S. maximus*) and has been characterized previously [45]. It was routinely cultured in marine broth 2216E (Difco Laboratories, Detroit, MI, USA) for 24 h at 28 °C. Frozen stocks were preserved at −80 °C in marine broth 2216E containing 15% (*v*/*v*) glycerol.

### 4.4. Vaccine Preparation

*V. anguillarum* MN was grown in marine broth 2216E in a 50 L fermenter at 28 °C. After reaching the stationary phase with a concentration of about 2 × 10^9^ cells/mL, the bacteria were inactivated by adding 0.1% (*v*/*v*) formalin at 4 °C for 48 h. The sterility and toxicity of the bacterin preparation were evaluated by the quality-control protocols described by Collado *et al.* [46].

### 4.5. Vaccination Procedure

Twelve groups of fish containing 180 fish each group were used for vaccination. As shown in Table 2, fish in groups 1–4 were first immersed for 10 min in seawater containing QSS (5, 25, 45, and 65 mg/L, respectively), then transferred into the bacterin suspension (1 × 10^8^ cell/mL) for bath vaccination for 30 min. As negative controls, fish in groups 5, 6, 7, and 8 only received 10 min immersion in various QSS solutions (5, 25, 45, and 65 mg/L, respectively). Fish in group 9 were immersed for 30 min in inactivated bacterin suspension (1 × 10^8^ cell/mL) without pre-treatment in QSS. Fish in group 10 were vaccinated with IP injection of 0.1 mL of inactivated bacterin (1 × 10^8^ cell/mL in PBS); Group 11 was injected with 0.1 mL of PBS served as the control for group 10; fish in group 12 without any treatment were used as blank control.

### 4.6. Challenges

On days 7, 14 and 28 post-vaccination, 30 fish from each group (vaccinated and controls) were challenged through bath or IP as described previously [46]. For bath challenges, fish were immersed in 20× LC50 of *V. anguillarum* (equivalent to 1 × 10^8^ CFU/mL in sterilized seawater) for 1 h with constant aeration at 17 °C. For IP injection challenge, fish were injected with 0.1 mL of 1× LD50 of *V. anguillarum* suspension (equivalent to 1 × 10^8^ CFU/mL in PBS). Fish mortalities were recorded daily for 15 days. Kidney, liver and skin from moribund fish were collected aseptically and analyzed to confirm the cause of mortality. The pure isolates of *V. anguillarum* MN from the internal organs of moribund fish were identified by slide agglutination with specific anti-*V. anguillarum* sera [47]. The efficacy of vaccination was evaluated with the relative percentage of survival (RPS), which was calculated by the following equation:

RPS = 1 − (% mortality in vaccinated fish/% mortality in controls) × 100
(1)

Three replicates were conducted in each challenge group.

### 4.7. Sample Collection

**Figure 4:**
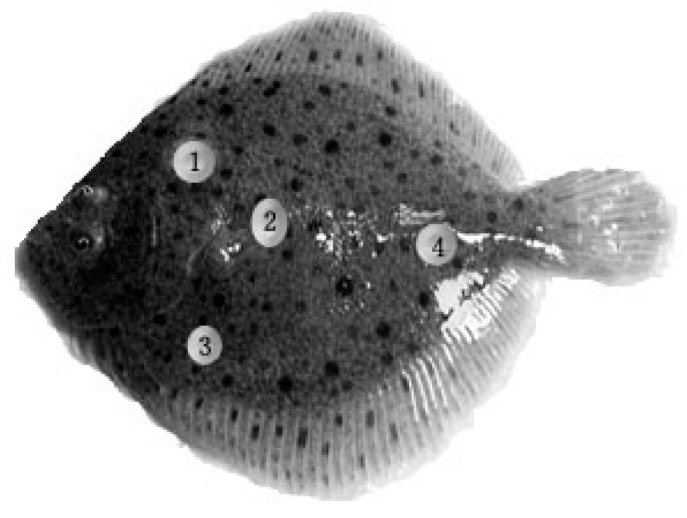
Sampling sites on the back side of turbot. Mark 1 was in the middle of the leading edge of dorsal fin and the trailing edge of operculum; Mark 2 was around the front end of lateral line; Mark 3 was at the place of one fourth from anus to point 2; Mark 4 was at the two thirds of lateral line.

All fish were starved for 24 h before sampling. A total of 9 fish were collected from each group of all vaccinated and control groups at days 0, 7, 14, 21 and 28 post-immunization. Blood was withdrawn by heparinized syringes from the caudal vein and used for serum preparation as described previously [41,46]. For mucus collection, fish were placed in empty sterile flask, and skin mucus was gently scraped using a soft rubber spatula and collected in a sterile Eppendorf tube containing PBS (1:1) with proteinase inhibitors (100×). Mucus samples were analyzed immediately. Subsequently, fish skin, gill, spleen and head-kidney were aseptically sampled. Briefly, skin tissues were collected from four sites (as shown in Figure 4) on the backside of the turbot (about 9 mm^2^ each), mixed together and kept at −80 °C till further use. Samples of fish gill, spleen and head-kidney (about 10–20 mm^3^) were cut off from each fish using a sterile surgical scissor and stored in RNA latter (Invitrogen, Carlsbad, CA, USA) for mRNA extraction.

### 4.8. Enzyme-Linked Immunosorbent Assay (ELISA)

Specific antibody titers (IgM) from sera of both immunized and non-immunized fish were assessed by ELISA in 96-well flat-bottom microtiter plates that were pre-coated with 0.05% poly-l-lysine (Corning-Costar Corp., Cambridge, MA, USA) as previously described with minor modification [48]. Briefly, 100 μL·well^−1^ of *V. anguillarum* resuspended in a PBS buffer (pH 9.6) (1.3 × 10^8^ cell·mL^−1^) were added and incubated at 4 °C overnight. Then, to each plate, 50 μL·well^−1^ of 0.05% (*v*/*v*) gluteraldehyde were added and incubated for 20 min at 22 °C. After 3 washes with low salt wash buffer, the plate was blocked by adding 250 μL per well of 1% bovine serum albumin (BSA) for 2 h at 22 °C. Then, the double-diluted serum samples from each groups of both immunized and non-immunized fish were loaded into each well in three replicates (100 μL·well^−1^), and the plate was incubated at 4 °C for 18 h. After washing, 100 μL·well^−1^ of mouse anti-turbot IgM monoclonal antibody (Mab) was added to the plate and incubated for 60 min at 22 °C. After 5 washes, 100 μL·well^−1^ of 1/1000 diluted goat anti-mouse IgG-HRP in a conjugate buffer was added to the plate and incubated for 60 min at 22 °C. Finally, 100 μL·well^−1^ of TMB substrate was added for color development, and the reaction was then terminated with 2.0 M H_2_SO_4_. The absorbance was detected with a microplate reader (Thermo Fisher Scientific, Waltham, MA, USA) at 450 nm. Results were considered as positive if the OD value showed at least three times more than that of the control samples. The antibody titers were scored at the highest positive dilution. The averaged antibody titer (*G*) was calculated according to the formula:

(2) $$G=log_{10}^{-1}(\frac{\sum^{\text{​}}flog_{10}^{X}}{\sum^{\text{​}}f})$$

### 4.9. RNA Extraction

Total RNA was extracted from various tissues by using TransZol Up (TransGen Biotech., Beijing, China) according to the manufacturer’s instruction. Briefly, tissues were grinded in 1 mL of TransZol Up to lyse the cells and release the RNA. After standing at room temperature for 5 min, 0.2 volume of chloroform was added into the suspensions and followed by vigorous shaking. After incubation at room temperature for 3 min, the samples were centrifuged at 12,000× *g* at 4 °C for 15 min, and the clear upper phase containing RNA was carefully transferred to a fresh Eppendorf tube. Then, 0.5× volume of cold isopropanol was added to allow RNA precipitation. The extracted RNA samples were spun down and then washed in 1 mL of cold 75% ethanol by centrifugation at 12,000× *g* for 5 min at 4 °C. The pelleted RNA samples were air-dried, then re-suspended in 50 μL of diethylpyrocarbonate (DEPC)-treated water, and kept at −80 °C. The total concentration and purity of RNA was determined using spectrophotometry.

### 4.10. Reverse Transcription

For the reverse transcription reaction, one microgram of RNA was diluted in DEPC-treated water in a final volume of 20 μL. One microliter anchored Oligo(dT)18 primer (0.5 μg·μL^−1^) (TransGen Biotech., Beijing, China), 10 μL of 2× TS Reaction Mix, and 1 μL of *Trans Script* RT/RI Enzyme Mix were then added. The reaction mixtures incubated for 30 min at 42 °C and then terminated by heating 5 min at 85 °C.

### 4.11. Quantitative Real-Time PCR (qPCR)

PCR amplification was performed in a reaction volume of 25 μL according to the manufacturer’s instruction (*TransStrat*™ green qPCR SuperMix) (TransGen Biotech., Beijing, China) by using a Rotor-Gene DNA sample analysis system. Primers and related reaction conditions were listed in Table 3 and Table 4, respectively. A housekeeping gene to encode β-actin was amplified as a positive control. Threshold cycle (*C*_t_) values were exported to Microsoft Excel for further analysis. The comparative *C*_t_ method (2^−∆∆*C*t^ method) was used to determine the gene expression level profile [24]. The relative expression of target genes was normalized in comparison to that of β-actin gene. Fold units were calculated by dividing the normalized gene expression values of immunized tissues by the normalized expression values of the controls.

### 4.12. Statistical Analysis

Significant differences among groups of various treatments and controls regarding immune responses (antibody titer and IgM gene express) and protection (RPS) were estimated using two-factor analysis of variance under SPSS16.0 Data Editor followed by the Duncan’s multiple range tests for comparing the means. Significant difference was considered at *p* < 0.05.

## 5. Conclusions

In conclusion, the vaccination strategy in our present study offered an excellent efficacy for turbot against *V. anguillarum* infection by combining inactivated *V. anguillarum* bacterins immersion with adjuvant QSS at the optimal subtoxic dose of 45 mg/L. This strategy, which not only induced significantly higher humoral antibody responses, but also elicited faster and remarkably high IgM gene expressions in skin, gill, spleen and kidney, was able to generate an equivalent protective effect to that of IP injection. These results conclusively indicated that QSS at a proper concentration might be a promising adjuvant candidate applying in fish vaccination via an immersion administering route.

## Figures and Tables

**Table 1:** Cumulative mortality of turbot challenged with *V. anguilarum* at days 7, 14 and 28 post-immunization *.

Groups	Cumulative Mortality (%)		
	7 Days	14 Days	28 Days
QSS5 + V	50.0 ± 10.0 ^b,c,d^	40.0 ± 10 ^c,d^	30 ± 26.5 ^c^
QSS5	60.0 ± 10.0 ^a,b,c^	63.3 ± 11.5 ^a,b^	60.0 ± 0 ^a,b^
QSS25 + V	46.7 ± 15.3 ^b,c,d^	23.3 ± 11.6 ^d,e^	26.7 ± 15.3 ^c^
QSS25	63.3 ± 5.7 ^a,b,c^	56.7 ± 11.5 ^b,c^	60.0 ± 0 ^a,b^
QSS45 + V	30.0 ± 10.0 ^e,f^	13.3 ± 5.7 ^e,f^	13.3 ± 5.8 ^c^
QSS45	66.7 ± 5.7 ^a,b,c^	63.3 ± 11.5 ^a,b^	53.3 ± 5.7 ^b^
QSS65 + V	30.7 ± 5.8 ^d,e,f^	20.0 ± 0 ^e,f^	26.7 ± 5.8 ^c^
QSS65	60.0 ± 10.0 ^a,b,c^	53.3 ± 20.8 ^b,c^	50.0 ± 10.0 ^b^
BI-V	53.3 ± 5.8 ^b,c,d^	40.0 ± 10.0 ^c,d^	26.7 ± 5.7 ^c^
Seawater	73.3 ± 15.3 ^a^	70.0 ± 10.0 ^a,b^	60.0 ± 10.0 ^a,b^
IP-V	20.0 ± 10.0 ^f^	3.3 ± 5.7 ^f^	10.0 ± 10.0 ^c^
PBS	43.3 ± 15.3 ^c,d,e^	80.0 ± 10.0 ^a^	76.7 ± 5.8 ^a^

PBS: Phosphate-buffered saline, BI: bath immersion, IP: intraperitoneal injection, V: vaccination; * Data represent the mean value ± S.E. of three replicates. Significant differences (*p* < 0.05) among groups were indicated by different letters.

**Table 2:** Experimental design for vaccination.

Groups	Way of Immunization	No. of Fish (No. of Replicates)	Dosage of Vaccine (cfu/mL)
QSS5 + V	BI	90(2)	1 × 10^8^
QSS5	BI	90(2)	0
QSS25 + V	BI	90(2)	1 × 10^8^
QSS25	BI	90(2)	0
QSS45 + V	BI	90(2)	1 × 10^8^
QSS45	BI	90(2)	0
QSS65 + V	BI	90(2)	1 × 10^8^
QSS65	BI	90(2)	0
BI-V	BI	90(2)	1 × 10^8^
Seawater	BI	90(2)	0
IP-V	IP	90(2)	1 × 10^8^
PBS	IP	90(2)	0

PBS: Phosphate-buffered saline, BI: bath immersion, IP: intraperitoneal injection, V: vaccination.

**Table 3:** Sequences of oligonucleotide primers used in qPCR reactions.

Gene	Primers Used	Sequence(5′→3′)	Location on Partial Sequence
β-actin	β-actin F	AAGCTGTGCTGTCCCTGTATG	311–331
β-actin	β-actin R	GCAGTGGTGGTGAAGGAGTAG	492–512
IgM	IgM F	TCAGTATCGACTTAGACACTTGCAG	70–94
IgM	IgM R	TCCCCAGTAGTCAAAGATCCAC	169–191

Accession numbers: β-actin: AY008305 [49]; IgM: AJ296096 [50].

**Table 4:** Summary of conditions used in qPCR amplification.

Target Gene	Composition of Reaction Mixture (μL)						Cycling Protocol				
	cDNA (μL)	Forward Primer (10 μM)	Reverse Primer (10 μM)	*TransStrat™* Green qPCRSuper Mix (2×)	Passive Reference Dye (50×)	Sterile Water	Denature	Anneal	Elongate	No. of Cycles	Product Size (bp)
β-Actin	2	0.5	0.5	12.5	0.5	9	95 °C/30 s			1	202
							95 °C/5 s	55 °C/15 s	72 °C/20 s	40	
IgM	2	0.5	0.5	12.5	0.5	9	95 °C/30 s			1	122
							95 °C/5 s	55 °C/15 s	72 °C/20 s	40	
